# *capD* deletion in the *Elizabethkingia miricola* capsular locus leads to capsule production deficiency and reduced virulence

**DOI:** 10.1186/s13567-024-01394-8

**Published:** 2024-11-11

**Authors:** Ruixue Hu, Fangyuan Liu, Fang Yu, Jiahao Hou, Dan Chen, Zemao Gu

**Affiliations:** 1 College of Fisheries/Shuangshui Shuanglü Institute, Huazhong Agricultural University, Wuhan, 430070 China; 2 Hubei Engineering Technology Research Center for Aquatic Animal Disease Control and Prevention, Wuhan, 430070 China; 3 National Aquatic Animal Diseases Para-reference Laboratory (HZAU), Wuhan, 430070 China

**Keywords:** *Elizabethkingia miricola*, capsule polysaccharide, *capD*, virulence

## Abstract

*Elizabethkingia miricola* is a multidrug-resistant pathogen that can cause life-threatening infections in immunocompromised humans and outbreaks in amphibians. However, the specific virulence factors of this microorganism have not been described. In this study, we identified the polysaccharide biosynthesis protein-encoding gene *capD*, which is located in the conserved region of the Wzy-dependent capsule synthesis gene cluster in the *E. miricola* strain FL160902, and investigated its role in the pathogenesis of *E. miricola*. Our results revealed that the *capD* deletion strain (Δ*capD*) lost its typical encapsulated structure, with a 45% reduction in cell wall thickness. CapD affects *wza* expression in the capsule polysaccharide synthesis pathway. Furthermore, the survival rates were significantly reduced in Δ*capD* in response to complement-mediated killing, desiccation stress, and macrophage phagocytosis, whereas biofilm formation, surface hydrophobicity, and adherence to both endothelial and epithelial cells were increased. Additionally, the deletion of *capD* sharply attenuated the virulence of *E. miricola* in a frog infection model. Complementation of the *capD* gene restored the biological properties and virulence to wild-type levels. Overall, these findings suggest that CapD contributes to polysaccharide synthesis and plays a crucial role in the pathogenesis of *E. miricola*.

**Supplementary Information:**

The online version contains supplementary material available at 10.1186/s13567-024-01394-8.

## Introduction

The *Elizabethkingia* genus is a group of Gram-negative, non-motile aerobic rods that are ubiquitous in natural and healthcare settings [1–3]. This genus includes seven species, such as *Elizabethkingia meningoseptica*, *Elizabethkingia miricola*, and *Elizabethkingia anophelis*, along with four additional novel species defined in recent years [4, 5]. Since its first identification in 1959, *Elizabethkingia* has emerged as a cause of life-threatening infections in humans characterized by severe clinical manifestations and high mortality rates, especially among neonates or immunocompromised individuals [6–9]. Certain species, such as *E. miricola*, have been associated with healthcare-associated outbreaks and have emerged as major causes of meningitis-like disease in the frog industry in China, leading to significant economic losses [10–12]. Additionally, *E. miricola* has been documented as an infectious pathogen in pet and wild frogs, posing a potential threat to public health and amphibian biodiversity [13]. Infections caused by *E. miricola* in frogs are characterized by clinical signs such as corneal opacity, ascites, and neurological manifestations. Notably, the pathogen is predominantly isolated from vital organs, including the brain, liver, spleen, and kidney [12, 13]. The treatment of *Elizabethkingia* infection is challenging because of its intrinsic multidrug resistance. Hence, understanding the mechanisms of its pathogenicity is crucial for devising effective strategies to prevent and control infections caused by *Elizabethkingia* [14, 15].

Studies on the pathogenicity of *Elizabethkingia* have identified various putative virulence mechanisms associated with biofilm formation, capsule polysaccharide synthesis (CPS), iron siderophore synthesis, superoxide dismutase expression, prevention of phagocytosis, inhibition of complement-mediated opsonophagocytosis, and other functions [16, 17]. Although these studies have enhanced our understanding of *Elizabethkingia* virulence, the identified virulence genes have been annotated solely on the basis of sequence homology to known reference sequences; thus far, functional characterization of key virulence factors is lacking.

Capsule polysaccharides are associated with virulence in various bacteria, facilitate evasion or neutralization of host immune responses, and play a pivotal role in biofilm formation and surface colonization [18, 19]. The potential contribution of capsule polysaccharides to the pathogenicity of *Elizabethkingia* has been investigated through genomic analysis of the CPS synthesis gene clusters in the strains involved in the Wisconsin and Singapore outbreaks. This analysis revealed that the outbreak isolates shared identical CPS gene clusters (type I), which have thus far been observed in only these two human *Elizabethkingia* strains, suggesting a possible pathogenic role for the capsular polysaccharide in the outbreak strains [8].

The production of *Elizabethkingia* capsule polysaccharides is dependent on the Wzy CPS biosynthesis pathway [3, 8]. The CPS gene cluster contains a highly conserved upstream region with the two well-characterized genes *wzc* and *wza*. These genes encode auxiliary proteins located in the inner membrane–periplasmic space and the outer membrane, respectively. Furthermore, this region also contains the conserved *capD* gene, which codes for a protein involved in polysaccharide biosynthesis [3]. The CapD protein has been confirmed as an important pathogenicity-associated determinant that contributes to resistance against complement-mediated killing in *Haemophilus parasuis* and bacterial colonization ability in *Enterococcus faecium* [20, 21]. However, its specific role during *Elizabethkingia* infection is not well understood.

In this study, we characterized the capsular gene clusters and *capD* gene of the *E. miricola* FL160902 strain, which was initially isolated from a diseased frog in China in 2016 [12]. To further elucidate the role of *capD* in capsule formation in *E. miricola* FL160902, we constructed *capD* knockout and complementation strains via homologous recombination. We subsequently assessed phenotypic changes, including changes in capsular growth, biofilm formation, desiccation resistance, hydrophobicity, serum resistance, and the ability to cause infection in vivo and in vitro. These results provide preliminary insights into the role of the *capD* gene in *E. miricola* infection, providing a basis for understanding the role of capsule polysaccharides in the virulence of *Elizabethkingia.*

## Materials and methods

### Bacterial strains, plasmids, reagents, and culture media

The bacterial strains and plasmids used in the present study are listed in Table 1. Bacterial culture was performed via brain–heart infusion agar medium (BHIA; Hopebio, Cat. No. HB0128) or Columbia sheep blood agar medium (CSBA; Hopebio, Cat. No. HBPM0153). The bacterial growth media were supplemented with antibiotics or sugars at specified concentrations: 100 µg/mL ampicillin (Amp, Biosharp, Cat. No. BS923), 100 µg/mL spectinomycin (Spc, Biosharp, Cat. No. BS169), 34 μg/mL chloramphenicol (Cm, Biosharp, Cat. No. C8050), 50 µg/mL erythromycin (Erm, Solarbio, Cat. No. E8100), and sucrose (SCR, Cat. No. 10021418), 10% w/v.

**Table 1:** **List of bacterial strains and plasmids used in this study**

Strains or plasmids	Description	Source or references
Strains		
FL160902 (WT)	*E. miricola* wild-type strain	Laboratory collection [12]
FL160902-Δ*capD*	*capD* deletion mutant of FL160902 strain, Spc^R^	This study
FL160902-CΔ*capD*	*capD* complemented mutant of FL160902 strain, Spc^R^	This study
*E. coli* S17-1 λ pir	RP4-2 (km::Tn7, Tc::Mu-1), LAMpir, recA1, hsdR17	WEIDI (Cat. No. DL2010)
Plasmids		
pRE112	Cm^R^, SacB suicide vector	[41]
pRE112-UD-spc^R^	Cm^R^ Spc^R^, suicide vector for *capD* deletion	This study
pYT354	Erm^R^, Amp^R^, SacB suicide vector	[23]
pYT354-UD-*capD*	Erm^R^, Amp^R^, suicide vector for *capD* complemented	This study

### Analysis of capsular gene clusters and CapD sequence homology

To identify capsular gene clusters in *E. miricola* FL160902 (GenBank accession no. GCA_009938095.1), we conducted a keyword search of the Pfam database for protein profiles involved in capsular polysaccharide production, including glycosyl transferases, ABC transporters, Wzx flippase, and Wzy polymerase [22]. We subsequently searched for these profiles in the genome of *E. miricola* FL160902 using HMMER3 (v.3.1b1), which has an E value < 10^−4^ and a protein coverage threshold of 50%. The amino acid sequence of the CapD protein was submitted to the National Center for Biotechnology Information (NCBI) nonredundant protein database under GenBank accession no. WP_078795750.1. Protein sequence comparison was carried out via BLASTP with CapD sequences from seven standard strains within the genus *Elizabethkingia*. ClustalW and GeneDOC were used for multiple sequence alignment.

### Construction of the *capD* knockout mutant and its complement strain

Homologous recombination was used to generate *capD* mutants. The primers used in this study are listed in Table 2. The upstream region (1162 bp) and downstream region (1077 bp) of the *capD* gene were amplified by PCR from the genome of *E. miricola* FL160902 using the primers *capD*-Up-F/R and *capD*-Down-F/R, respectively. The *spc*^R^ gene was amplified from the pIC333 plasmid using primers *spc*-F/R. The suicide vector pRE112 was digested with SacI (Takara, Code No. 1627) and XbaI (Takara, Code No. 1634) restriction endonucleases to obtain vector fragments. The resulting PCR fragments upstream fragment, *spc*^R^ PCR fragment, upstream fragment, and pRE112 plasmid fragment were integrated using the ClonExpress MultiS One Step Cloning Kit (Vazyme, Cat. No. C113-01) to generate the knockout plasmid pRE112-UD-spc^R^. The donor strain *E. coli* S17-1 λpir carrying the pRE112-UD-spc^R^ plasmid and the recipient strain *E. miricola* FL160902 were cultured overnight and adjusted to a cell density of 1 × 10^8^ CFU/mL. Samples of the two strains were mixed and washed three times with 10 mM MgSO_4_ solution. The mixture was dropped onto a millipore filter membrane (0.22 μm) and incubated on BHI agar at 37 ℃ for 24 h. Transconjugants with the first recombination insertion on the FL160902 chromosome were selected on BHI plates containing Cm, Spc, and Amp. The putative transformants were confirmed by PCR using a series of specific primers.

**Table 2:** **List of primers used for PCR and DNA sequencing**

Primers		Sequence (5′–3′)	Tage gene
*spc*-F		TTCGTATGCCGTCTTCTG	*Spc*^R^
*spc*-R		CTGGCAGTTCCCTACTCT	
*capD*-Up-F		ACTGCATGAATTCCCGGGAGTCAAGAACGATGAGGTGT	Upstream fragment of *capD*
*capD*-Up-R		CAGAAGACGGCATACGAAAACGCTCGCTGTAAATAG	
*capD*-Down-F		AGAGTAGGGAACTGCCAGAAAATGATAGTGCCAGAG	Downstream fragment of *capD*
*capD*-Down-R		CGATCCCAAGCTTCTTCTAGGCAATAACTAATGCCAAG	
neutral site-Up-F		TACTCAGAGCTCGGTGGAGCACCTATGT	Upstream fragment of neutral site
neutral site-Up-R		TACTCAGGATCCTCGTATGAGGGCTCTT	
neutral site-Down-F		TACTCAGTCGACGGGCTCTGTAATAATTCGGG	Downstream fragment of neutral site
neutral site-Down-R		TACTCAGGTACCGGAACCCCTGCTCAAA	
PompA-F		AGCCCTCATACGAGGATCCCTTGCCACATTTGGTG	*ompA* gene promoter
PompA-R		CAGTTCTTCGCCTTTGCTC	
*capD*-F2		GAGCAAAGGCGAAGAACTGCTGCCAAGGTGGATTGTTCT	Full-length sequence of *capD*
*capD*-R2		TTATTACAGAGCCCGTCGACGGAGTGCTGTTTCGGTAGCT	
*capD*-F		AATCCTAGCCTGCCTTAT	Internal sequence of *capD*
*capD*-R		AGTTGACGCACTACTTCT	
Check-knockout-F		ACGTAGGATTTACTCTGG	External sequence of *capD*
Check-knockout-R		AACAACTGACCTGATGAG	
Check-knockin-F		GATAGAACTTGCAGACGG	External sequence of neutral site
Check-knockin-F		TTCAGAGTAGTGCACAGG	

The gene complementation strain was constructed by integrating the *capD* gene into the Δ*capD* strain chromosome as previously described [23]. A region was selected as a neutral site for gene integration into the chromosome via homologous recombination. This region, which is highly conserved and located downstream of the *plc* gene, is not associated with the virulence of *E. miricola* FL160902 (data not shown). The upstream region (2125 bp) and downstream fragment (2018 bp) of the neutral site were amplified by PCR from the genome of *E. miricola* FL160902 using the primers neutral site-Up-F/R and neutral site-Down-F/R, respectively. The *capD* gene was amplified from the genome of *E. miricola* FL160902 using primers *capD*-F2/R2. The *ompA* gene promoter fragment (114 bp) was artificially synthesized by Shenggong Biotechnology Co., Ltd. (Shanghai, China). The suicide plasmid pYT354 was digested with Kpn I (Takara, Code No. 1618) and Sac I to generate a linear vector. Afterward, the upstream fragment, *ompA* promoter fragment, *capD* gene fragment, and downstream fragment were ligated with the linear plasmid pYT354 using the ClonExpress MultiS One Step Cloning Kit to generate the knock-in plasmid pYT354-UD-*capD*. The knock-in plasmid was introduced into the Δ*capD* strain by conjugation, using homologous recombination in a method similar to that used for gene knockouts.

### Detection of the genetic stability of the strains and growth curves

The Δ*capD* and CΔ*capD* (*capD* complementation strain) strains were passaged more than 30 consecutive times in BHI media at 37 °C. The genetic stability of the Δ*capD* and CΔ*capD* strains was assessed by PCR using the primers *capD*-F/*capD*-R. To measure the growth rate of the Δ*capD* strains, overnight bacterial cultures were transferred at a ratio of 1:100 into fresh preheated BHI, and growth was monitored at 37 °C with shaking using a FLUOstar Omega spectrophotometer (BMG Labtech). The mean optical density at 600 nm (OD_600_) values were determined every 1 h for 24 h, and the growth rates were estimated using GraphPad software.

### RT-qPCR measurement of *wza*, *wzc*, *wzy* and *capD* expression

To demonstrate the expression of crucial genes in the *capD* neighborhood of the capsule polysaccharide synthesis gene cluster of *E. miricola* FL160902, overnight bacterial cultures of the wild-type, ∆*capD*, and CΔ*capD* strains were centrifuged. Total RNA was extracted using TRIzol reagent (Cat. No. R401-01) according to the manufacturer’s instructions. RNA was reverse-transcribed using a reverse transcription kit (TaKaRa) to obtain cDNA, which was subsequently used for real-time RT-PCR (Applied Biosystems QuantStudio 6 Real-time PCR System, Thermo Fisher, Shanghai, China). The expression levels of *wza*, *wzc*,* wzy* and *capD* were determined based on the results of RT-qPCR, with 16S rRNA used as an internal reference. The primer sequences are listed in Additional file 1. The relative levels of target gene expression were normalized to those of 16S RNA using the 2^−∆∆Ct^ method.

### Capsule staining and transmission electron microscopy (TEM)

The *E. miricola* capsule was identified using light microscopy, followed by negative staining with India ink. The bacterial cells were washed with sterile PBS three times and resuspended in PBS. The washed bacterial suspension was mixed with India ink on a glass slide, spread thinly, and air dried. Fixation was performed with 95% ethanol, followed by counterstaining with 1% crystal violet. The presence of capsule polysaccharides was evaluated via light microscopy (DP73, Olympus, Japan).

A single colony of *E. miricola* was selected from Columbia sheep blood agar plates and cultured in BHI liquid medium supplemented with 10% fetal bovine serum. Following centrifugation of a 3 mL overnight culture, the cells were washed three times with 0.1 M phosphate-buffered saline (PBS, pH 7.4). After the supernatant was discarded, the bacteria were fixed with 2.5% glutaraldehyde (Sangon Biotech, Shanghai, China) and incubated at 4 °C overnight. After three washes with 0.1 M PBS and fixation with 1% citrate fixative for 1 h, the cells were dehydrated for 20 min with gradient acetone. An agent and acetone were sequentially introduced for gradient embedding, followed by sample mixing with the pure agent. The samples were sliced using an ultrathin trimming machine and observed using a transmission electron microscope (TEM, HITACHI H-7000FA, Hitachi Scientific Instruments, Japan). The thickness of the bacterial capsule was determined via ImageJ software by randomly selecting 30 cells.

### Biofilm formation and hydrophobicity test

Biofilm production was detected using a microtiter assay with minor modifications [24]. The wild-type, ∆*capD*, and C∆*capD* strains were cultured in BHI broth at 37 °C with rotation at 200 rpm overnight and then adjusted to a cell density of 1 × 10^8^ CFU/mL. Two hundred microliters of a 1:100 dilution of bacterial cultures was dispensed into polystyrene flat-bottom 96-well plates (JET BIOFIL, Cat. No. TCP011096) and incubated at 37 °C for 72 h. Each sample was incubated in quadruplicate, and BHI broth was used as a negative control in each plate. The bacterial culture mixture was removed, and the wells were washed three times with PBS (pH = 7.4). The biofilms were fixed at 60 °C for 1 h and stained with a 0.5% crystal violet solution (Cat. No. G0165). After 15 min, the staining solution was discarded, and the wells were rinsed three times with deionized water to remove excess stain. The plates were air-dried for 1 h, and the crystal violet dye bound to the biofilm cells was solubilized in 200 µL 95% ethanol for 15 min. After solubilization, 180 µL of the solution was transferred to a new plate, and the absorbance was measured at 595 nm via a microplate reader (TECAN, Nano Quant Infinite M 200 PRO).

The hydrophobicity assay was performed as follows. Briefly, the wild-type, ∆*capD*, and C∆*capD* strains were cultured to the logarithmic growth phase, washed three times, and adjusted to a cell density of 1 × 10^8^ CFU/mL in PBS. Six millilitres of PBS-suspended cells in a glass tube were thoroughly mixed with 2 mL of octane for 2 min, after which the initial OD_600_ value (ODI) was measured. The OD_600_ values of the aqueous phase were subsequently measured after 30 min (ODF). The percentage of cell hydrophobicity was calculated as follows: [1 − (ODF/ODI)] × 100%.

### Desiccation test

The desiccation viability of the wild-type, ∆*capD*, and C∆c*apD* strains was assessed as follows. The bacterial suspensions were incubated on polycarbonate membranes placed on BHI agar at 37 °C for 18 h. The cells on the membranes were resuspended and counted after being subjected to desiccation for 30 and 60 min at 37 °C. The samples were diluted 10-fold, spread on plates, and incubated overnight, after which the colonies were counted. The survival rate was calculated via the following formula: bacterial survival rate = (viable count after drying/viable count before drying × 100%). All the assays were repeated three times.

### Complement-mediated killing test

The susceptibility of the wild-type, ∆*capD*, and C∆*capD* strains to complement-mediated killing was determined as described previously, with a few modifications [20]. Ten milliliters of blood were collected from healthy frogs and centrifuged at 3000 rpm to obtain normal serum (NS). The pooled serum samples were filter sterilized (0.22 μm), and aliquots were stored at −80 °C. Aliquots were placed in a water bath at 56 °C for 30 min to inactivate the complement system, generating heat-inactivated serum (HS). The wild-type, ∆*capD*, and C∆*capD* strains were grown to the mid-log growth phase in BHI broth and resuspended in phosphate-buffered saline (PBS) to a cell density of 1 × 10^7^ CFU/mL. We measured serum resistance at 50% and 90% serum concentrations and incubated the samples at 28 °C for 30 min. The samples were diluted tenfold, spread onto plates, incubated overnight, and colonies on the plates were counted. The bacterial survival percentage was calculated by dividing the number of surviving bacteria treated with normal serum (NS) by the number of surviving bacteria treated with heat-inactivated serum (HS).

### Bacterial adhesion and anti-phagocytosis assays

Bacteria cultured overnight in BHI medium were pelleted, resuspended, and diluted in warm serum-free DMEM to a cell density of 10^8^ CFU/mL for infection. Adhesion experiments were performed with bEnd.3 cells (a mouse brain endothelial cell line) and EPCs (epithelioma papulosum cyprini cell line). Briefly, bEnd.3 cells were incubated in 12-well tissue culture plates to 80–90% confluence and infected with the wild-type, ∆*capD*, or C∆*capD* strains at an MOI of 20. The infected cells were incubated at 37 °C for 2 h. Nonadherent cells were washed with sterile PBS three times, and the infected cells were lysed with 1.0% (v/v) Triton X-100 for 10 min. The number of cell-adherent bacteria was determined after tenfold dilution and spread onto BHI plates. The EPC cell adhesion experiment was performed as described above but at 28 °C without CO_2_ and in M199 medium.

Anti-phagocytosis assays were performed with RAW264.7 cells (a murine monocyte macrophage line) in the same way as the adherence assay, with minor modifications. RAW264.7 cells were incubated in 6-well tissue culture plates and infected with a bacterial suspension at an MOI of 20. The extracellular bacteria were further treated with 100 µg/mL gentamicin (DMEM-Gent) for 60 min, followed by washing with sterile PBS three times, prior to lysis. The presence of intracellular bacteria was then determined as described for the adhesion experiments above.

### Experimental infection of frogs

To compare the pathogenicity of the wild-type, ∆*capD*, and C∆*capD* strains, a bacterial challenge experiment was performed using a previously described frog model [12]. The healthy black spotted frogs (*Pelophylax nigromaculata*) (weighing 25 ± 0.5 g) used in the present study were purchased from a frog farm (Qianjiang, Hubei, China). Overnight cultures of the strains were washed three times with sterile PBS and adjusted to the same cell density of 1 × 10^8^ CFU/mL. Healthy frogs were divided into three groups, each consisting of 20 frogs, and infected with 100 µL of the wild-type, ∆*capD*, or C∆*capD* strains via intramuscular injection. Negative controls were treated with equal volumes of aseptic PBS. The frogs were monitored for clinical signs of torticollis and cataracts for 2 weeks, and dead frogs were recorded. Brain, liver, and kidney samples were collected to confirm the cause of death.

To test the ability of bacteria to colonize within tissues, the same infection experiment was conducted. At 5 days post-infection, three frogs from each tank were arbitrarily selected and euthanized, and the bacterial loads in the liver, kidney, spleen, and brain homogenates were determined under aseptic conditions [25]. The logarithm of the bacterial load, expressed in copies per milligram, was used as the unit of measurement. All animal experiments were approved by the Laboratory Animal Monitoring Committee of Huazhong Agricultural University (202404280005) and performed in accordance with the recommendations of the ARRIVE guidelines.

### Statistical analysis

The data are shown as the mean ± SD and were analysed by one-way ANOVA or Student’s *t* test using GraphPad Prism (GraphPad Software, California, USA). The survival curves in the virulence assay were analysed using the log-rank test in GraphPad Prism. Statistical significance was set at *P* < 0.05. Each experiment was performed in triplicate and independently repeated three times.

## Results

### In silico characterization of the capsular gene clusters and *capD* in the *E. miricola* genome

**Figure 1:**
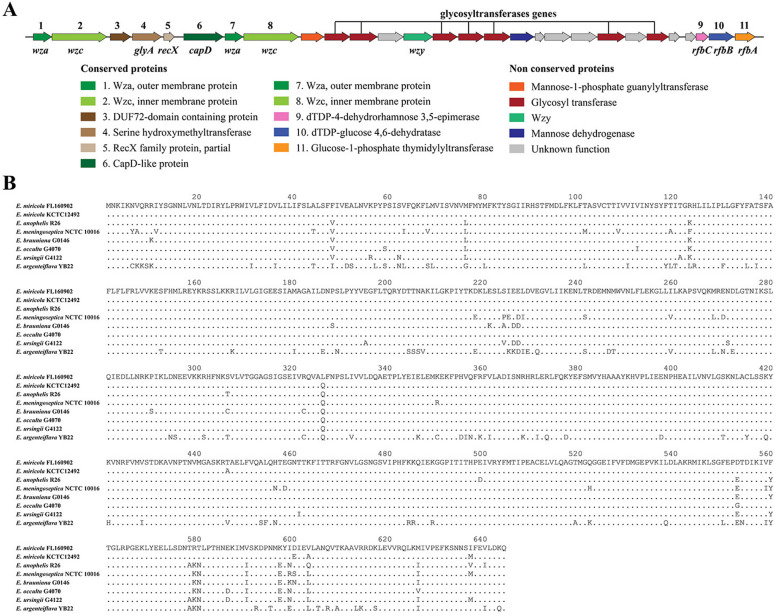
**Genetic organization of the CPS synthesis cluster in** ***E. miricola*** **FL160902 and multiple alignments of CapD.** **A** Genetic organization of the putative capsular polysaccharide synthesis protein cluster in *E. miricola* FL160902. Open reading frames (ORFs) are shown as arrows and are drawn to scale; intergenic spaces are not to scale. The numbers on top of the first genome correspond to the conserved proteins, and their annotations are detailed at the bottom of the figure. **B** Multiple sequence alignment of CapD from seven *Elizabethkingia* strains. The dotted lines indicate amino acid identity with *E. miricola* FL160902 CapD. The accession numbers of CapD proteins are WP_078795750.1 in *E. miricola* FL160902, WP_065081860.1 in *E. miricola* KCTC12492, WP_009087013.1 in *E. anophelis* R26, WP_016200520.1 in *E. meningoseptica* NCTC 10,016, WP_078674683.1 in *E. bruuniana* G0146, WP_078772978.1 in *E. occulta* G4070, WP_059325276.1 in *E. ursingii* G4122, and WP_166519632.1 in *E. argenteiflava* YB22.

A Wzy-dependent capsule synthesis gene cluster comprising 28 co-oriented coding sequences was identified in *E. miricola* FL160902 (Figure 1). The *capD* gene, which is involved in polysaccharide biosynthesis and is located in the conserved upstream (5′) part of the gene cluster, has been identified alongside two well-characterized capsular export genes, *wza* and *wzc*. The middle of the gene cluster contains a highly variable region encoding proteins involved in generating the specific polysaccharide composition of the capsule, including *wzy* (polymerase), as well as genes encoding glycosyl transferases, mannose dehydrogenase, and other sugar-modifying enzymes. The specific information is listed in Additional file 2. Bioinformatic analysis revealed that the *capD* gene consists of 1935 base pairs and encodes 644 amino acids. Furthermore, multiple sequence alignment revealed that CapD is a conserved protein within the genus *Elizabethkingia*, with homology ranging from 97.67% in *E. occulta* to 94.1% in *E. meningoseptica*, whereas the CapD of *E. argenteiflava* exhibited only 83.39% similarity.

### Identification of *capD* gene deletion and complementation

**Figure 2:**
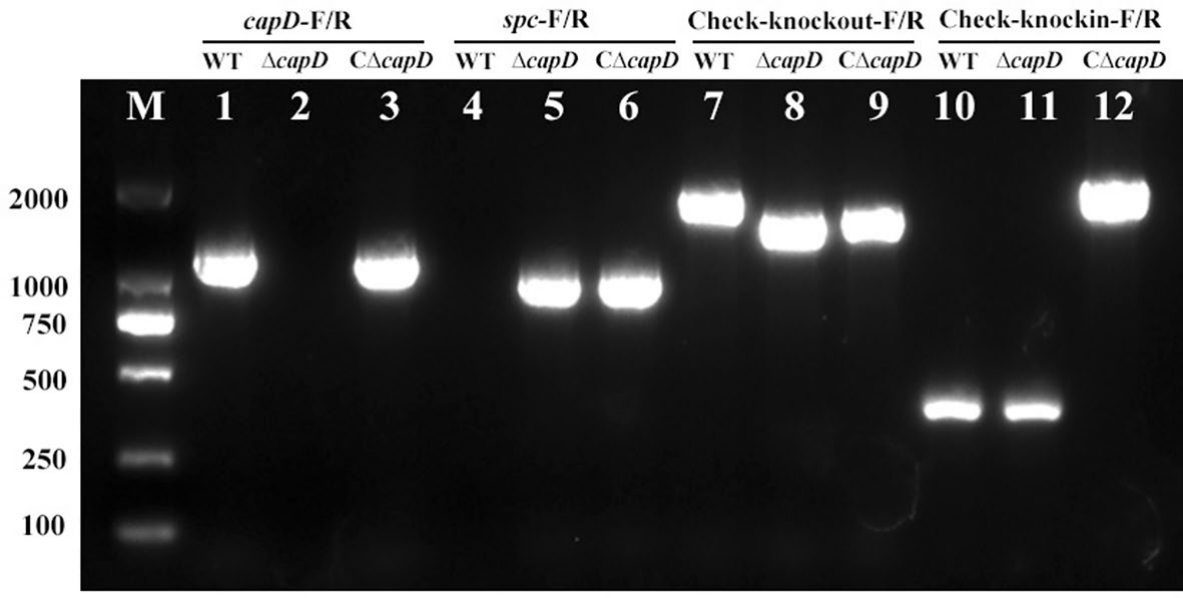
**Identification of the** ***E. miricola*** **FL160902, ∆*capD,*** **and C∆*capD*** **strains by PCR.** M: DL 2000 DNA Marker; lanes 1–3: identification of the *capD* gene via the primers *capD*-F/R; lanes 4–6: identification of the *Spc*^R^ gene via the primers *spc*-F/R; lanes 7–9: identification of the external sequence of *capD* via the primers Check-knockout-F/R; lanes 10–12: identification of the external sequence of the neutral site using the primers Check-knockin-F/R.

**Figure 3:**
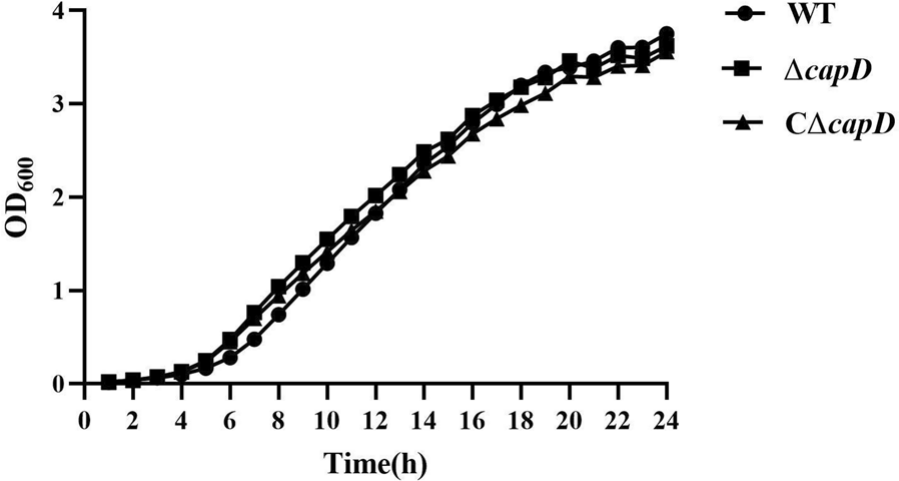
**Growth of** ***E. miricola*** **strains cultured at 37 °C in BHI broth.** The cell density was measured spectrophotometrically at a wavelength of 600 nm.

The polysaccharide biosynthesis gene *capD* was deleted in the *E. miricola* wild-type strain FL160902 by suicide plasmid-based homologous recombination, as illustrated in Figure 2. The *capD* fragment (*capD*-F/R) could not be amplified from the Δ*capD* strain (Lane 2), but the corresponding fragment was amplified from the wild-type and CΔ*capD* strains (Lanes 1 and 3, 1313 bp). The *Spc*^*R*^ fragment (*spc*-F/R) was amplified from the Δ*capD* and CΔ*capD* strains (Lanes 5 and 6, 1082 bp), whereas it was not amplified from the wild-type strain (Lane 4). These results indicate the successful replacement of the *capD* gene with the *Spc*^*R*^ gene in the Δ*capD* strain. Additionally, PCR amplification with *capD* gene external primers (Check-knockout-F/R) revealed that the amplification product of the Δ*capD* strain (Lane 8) differed from those of the wild-type and CΔ*capD* strains (Lanes 7 and 9), further confirming the deletion of the *capD* gene. PCR amplification using external primers at the insertion site (Check-knockin-F/R) yielded different amplification products for the CΔ*capD* strain (Lane 12) than for the wild-type and Δ*capD* strains (Lanes 10 and 11), indicating the successful integration of the *capD* gene into the Δ*capD* chromosome. The growth rates of the Δ*cap*D and CΔ*capD* strains were similar to that of the wild-type strain (Figure 3).

### *CapD* influences Wzy transport pathway in *E. miricola*

**Figure 4:**
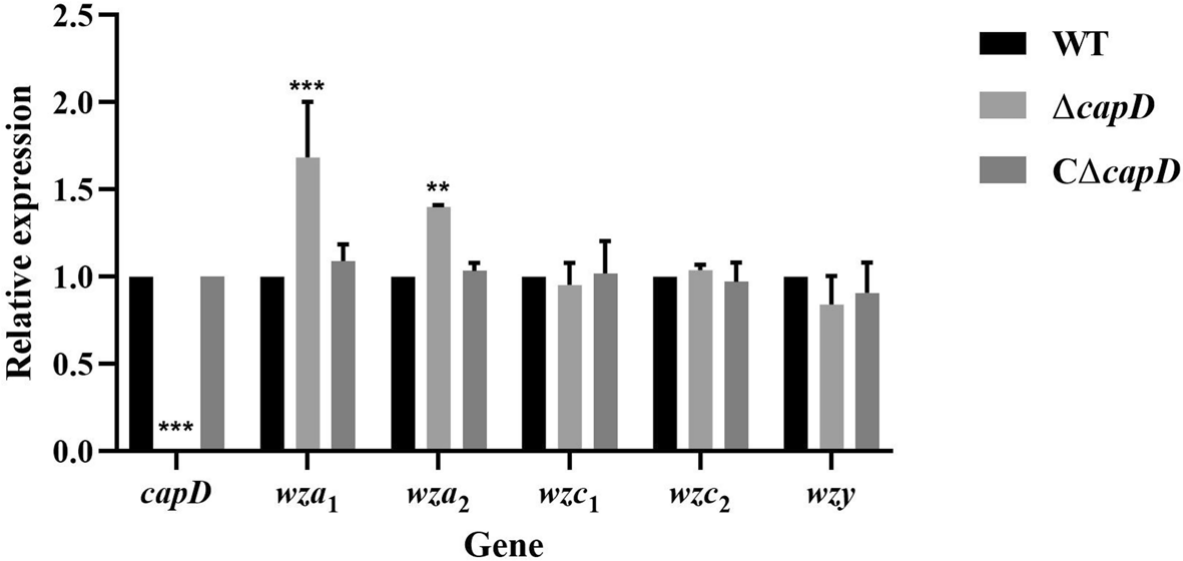
**Expression of** ***capD,*** ***wza,*** ***wzc,*** **and** ***wzy*** **in** ***capD*** **gene knockout and complementation strains.** Detection of the expression of six genes in strains by RT-PCR. The housekeeping gene 16S rRNA was used as an internal control, and the error bars represent the SEM of three replicates. ***Represents a significant difference at *P* < 0.001. Significant differences were determined by Student’s *t* test.

Analysis of the expression levels of genes located upstream and downstream of *capD* revealed that *wza*1 and *wza*2 were significantly increased by 1.69-fold and 1.40-fold, respectively, in the *capD* knockout strain compared with the wild-type strain (*P* < 0.001). Furthermore, the expression of these two genes was restored to wild-type levels in the *capD* complementation strain (Figure 4). These results indicate that the CapD protein affects the expression of *wza* in the Wzy transport pathway.

### *CapD* contributes to the morphology alteration induced by capsular polysaccharide formation

**Figure 5:**
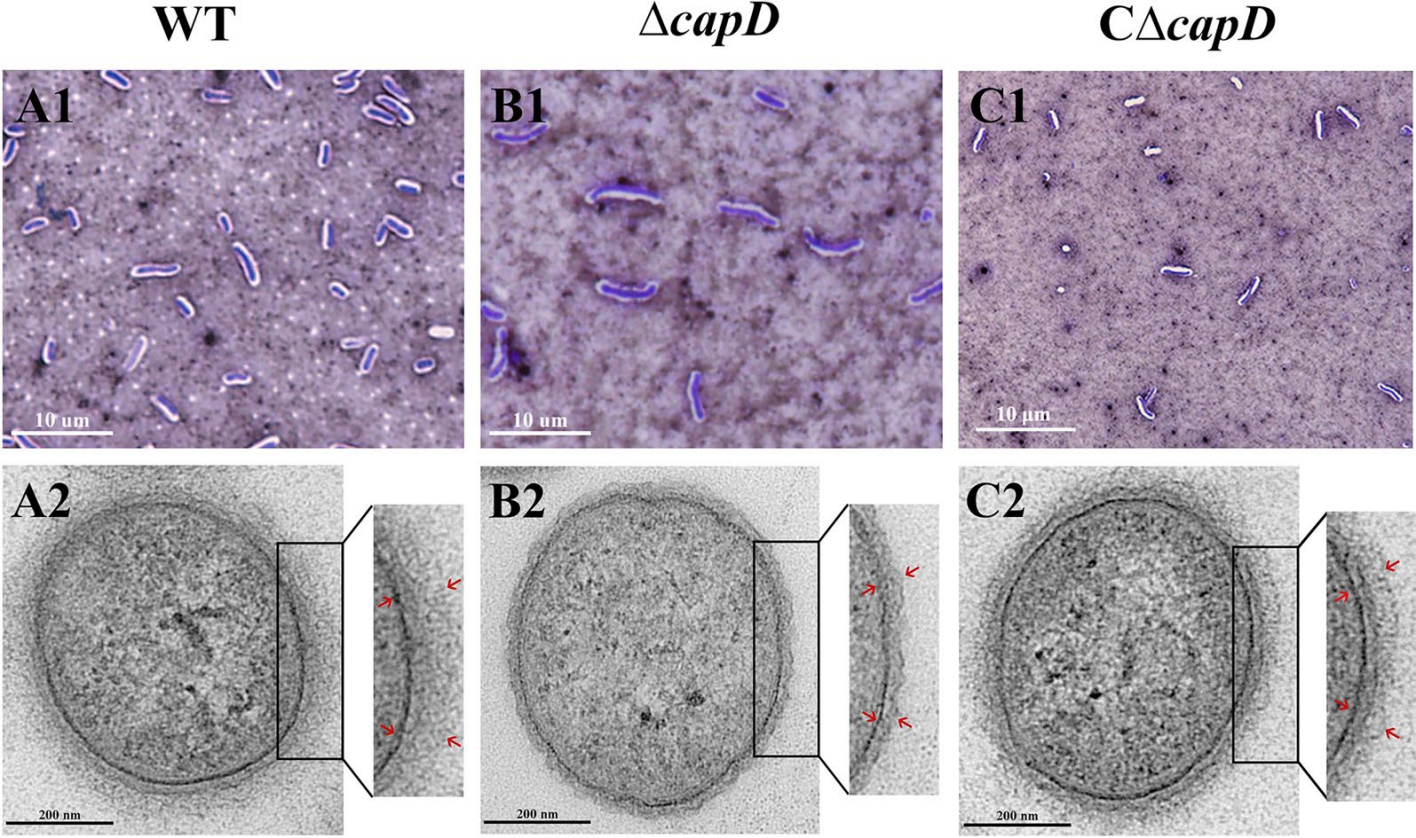
**Bacterial morphology by capsule staining and transmission electron microscopy.** Morphology of the wild-type strain (**A1**–**A2**), ∆*capD* mutant (**B1**–**B2**) and C∆*capD* strain (**C1**–**C2**). **A1**, **B1**, **C1** Observation of bacterial capsules by light microscopy (×1000) revealed that the polysaccharide capsule appeared as a clear halo around the microorganism in the WT strain (**A1**), the layer was thinned in the ∆*capD* mutant strain (**B1**), the complementation strain was recovered (**C1**), and the scale bar represents 10 μm. **A2**, **B2**, **C2 **Observation of bacterial capsules by transmission electron microscopy (×19000) revealed that the red tip showed a CPS layer approximately 40 nm thick in the wild-type strain (**A2**), the mutant strain expressed only a 22 nm thick CPS layer (*P* < 0.001) (**B2**), and the complementation strain expressed a 39 nm thick CPS layer (**C2**).

The modified Hiss capsule stain method was used to visualize the polysaccharide layer of the wild-type, Δ*capD*, and CΔ*capD* strains. Upon staining with crystal violet, the bacteria appeared dark purple, and the polysaccharides in the capsule replaced the colloidal carbon particles in the ink, forming a transparent halo around the microorganisms. The capsule sizes of the *E. miricola* wild-type and CΔ*capD* strains were slightly larger than those of the Δ*capD* strain (Figure 5A1, B1, C1). Further transmission electron microscopy (TEM) observations revealed that the cell wall of Δ*capD* appeared smoother and formed a denser capsule layer than did the wild-type strain. Additionally, as depicted in Figure 5, the cell wall thickness of the Δ*capD* strain was 22.52 nm (Figure 5B2), which was significantly less than that of the wild-type strain at 40.94 nm (Figure 5A2; *P* < 0.001). Furthermore, the thickness of the cell wall of the CΔ*capD* strain (39.14 nm) returned to that of the WT strain, and the cell surface recovered its rough appearance (Figure 5C2). Our findings indicate that knockout of the *capD* gene results in a deficiency in bacterial capsule production, highlighting the correlation between the *capD* gene and capsular polysaccharide formation.

### *CapD* contributes to anti-complement killing and desiccation tests in vitro

**Figure 6:**
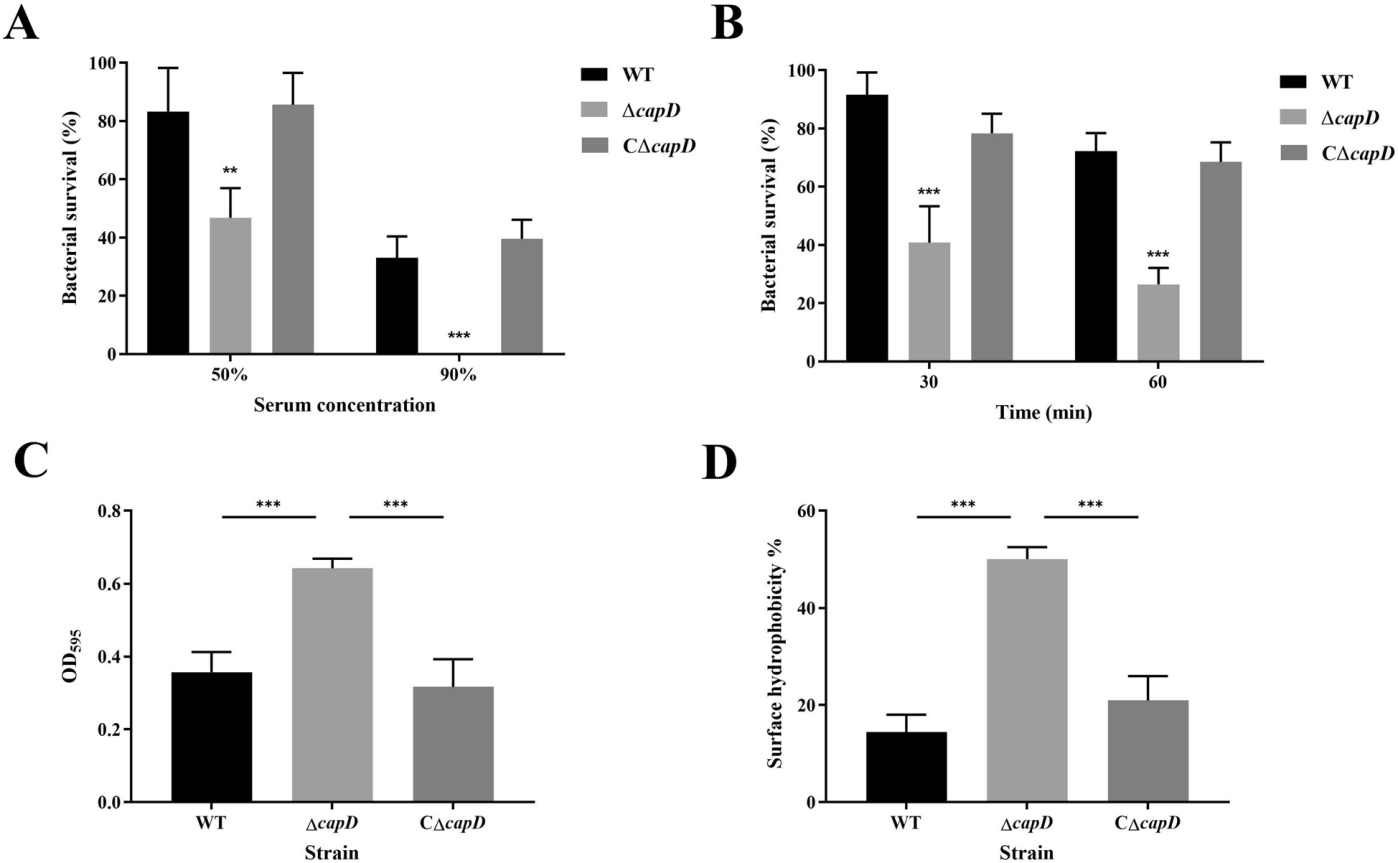
**Phenotypic characteristics of the** ***E. miricola*** **FL160902,** **Δ*capD,*** **and CΔ*capD *complementation strains.** **A** Survival percentages of the WT, Δ*capD* and CΔ*capD* strains in frog serum; **B** comparison of survival rates among the WT, Δ*capD* and CΔ*capD* strains under desiccation conditions; **C** biofilm formation capacities of the WT, Δ*capD* and CΔ*capD* strains; **D** percentages of cells in the hydrophobic solvent octane; error bars represent the mean ± standard error (*P < 0.05, ***P < 0.001).

To investigate the role of *capD* in mediating resistance against complement-mediated killing, the wild-type, Δ*capD*, and CΔ*capD* strains were incubated with either 50% or 90% frog serum. As shown in Figure 6A, the relative survival rate of the Δ*capD* strain was only 46.83% at a 50% serum concentration (*P* < 0.001), whereas that of the WT strain was 83.33%. No survival was observed for the Δ*capD* strain after incubation with 90% serum (*P* < 0.01). Compared with the WT strain, the *capD* mutant exhibited increased sensitivity to serum killing. No significant difference was observed in the survival rates between the CΔ*capD* strain and the wild-type strain, regardless of incubation with 50% or 90% serum, indicating that the serum resistance ability of the CΔ*capD* strain was restored. These results suggest that *capD* is involved in resistance to complement killing.

As shown in Figure 6B, the survival rates of the wild-type, Δ*capD*, and CΔ*capD* strains were determined under 37 °C desiccation conditions. After 30 min of drying, the Δ*capD* strain exhibited a survival rate of 40.82%, whereas the survival rate of the wild-type strain was 91.67%, resulting in a decrease in the survival rate. A similar trend was observed after 60 min of drying (Figure 6B).

### *CapD* prevents biofilm formation and surface hydrophobicity

Since biofilm production is often associated with extracellular polymers, we analysed biofilm formation in the *E. miricola* wild-type, Δ*capD*, and CΔ*capD* strains. The mean OD_595_ value of the Δ*capD* strain was 0.642, which was significantly greater than that of the wild-type strain (OD_595_ = 0.35; *P* < 0.01; Figure 6C). In comparison, the CΔ*capD* strain presented biofilm levels similar to those of the wild-type strain (OD_595_ = 0.31). These results indicate that the Δ*capD* strain exhibited a significant increase in biofilm formation capacity. We also compared the hydrophobic properties of the Δ*capD* strain with those of the wild-type and CΔ*capD* strains. As shown in Figure 6D, the surface hydrophobicities of the wild-type and CΔ*capD* strains were 14.42% and 50.08%, respectively, whereas the corresponding percentage for the Δ*capD* strain was 20.95%, demonstrating that the mutation led to a cell with a surface that was much more hydrophobic (*P* < 0.001). These results indicate that *capD* prevents the growth of bacterial biofilms and is involved in surface hydrophobicity.

### *CapD* affects adhesion and anti-phagocytosis ability in *E. miricola*

**Figure 7:**
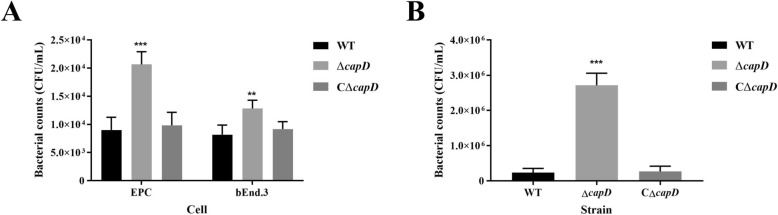
**Bacterial adhesion and anti-phagocytosis assays.** **A** The ability of WT, Δ*capD* and CΔ*capD* to adhere to EPC and bEnd.3 cells. **B** Anti-phagocytotic ability of WT, Δ*capD* and CΔ*capD* in RAW264.7 macrophages. The error bars represent the means ± standard deviations from three independent experiments (**indicates *P* < 0.01; ***indicates *P* < 0.001; Student’s *t* test).

The EPC and bEnd.3 cell lines were used as in vitro bacterial adhesion models. As shown in Figure 7A, compared with the wild-type strain (9.00 × 10^3^ CFU/mL), the *capD* mutant exhibited an approximately 2.3-fold increase in adherence (2.07 × 10^4^ CFU/mL, *P* < 0.001) in EPC cells. A similar difference in adhesion was observed with bEnd.3 cells (1.28 × 10^4^ vs. 8.17 × 10^3^ in Δ*capD* and wild-type cells, respectively). Furthermore, cell adhesion of the *capD* complementation strain reached the level of the WT strain in both cell types. We further compared the anti-phagocytic abilities of the wild-type, Δ*capD*, and CΔ*capD* strains in murine RAW264.7 macrophages. As depicted in Figure 7B, the bacterial count of the mutant strain was 2.75 × 10^6^ CFU/mL, which was approximately 11-fold greater than that of the wild-type strain (0.25 × 10^6^ CFU/mL). This finding indicated that the sensitivity of Δ*capD* to phagocytic cells increased compared with that of the WT strain, resulting in a significantly weakened anti-phagocytosis ability in Δ*capD* (*P* < 0.05). Compared with the mutant strain, the complemented strain presented a partially restored anti-phagocytosis ability. These results indicate that the *capD* gene can increase the ability of bacteria to adhere to EPC and bEnd.3 cells and attenuate phagocytosis.

### *CapD* contributes to virulence in a frog infection model

**Figure 8:**
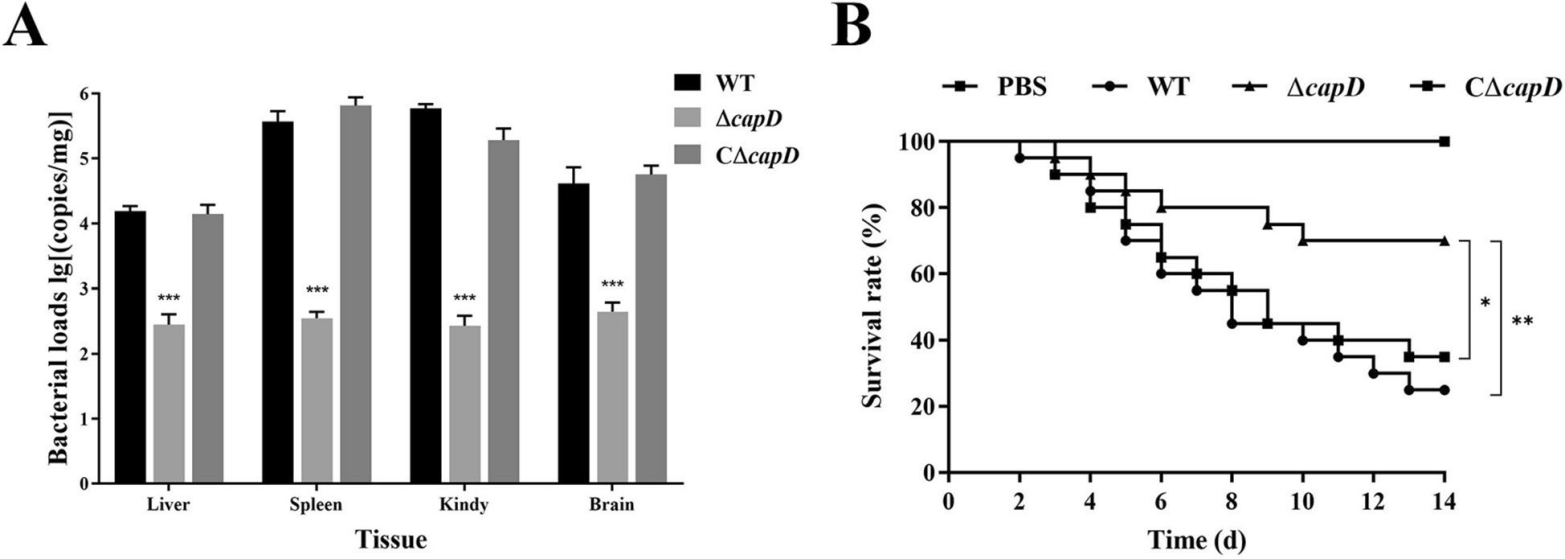
**Effects of** ***capD*** **on the pathogenicity of** ***E. miricola*** **in frogs.** **A **Colonization ability of frog tissues after challenge with WT, Δ*capD* or CΔ*capD*. Statistically significant differences are indicated as ***P < 0.001. **B **Kaplan‒Meier curve analysis of the survival of frogs after infection. Phosphate-buffered saline (PBS) was used.

A frog infection assay was used to determine the contribution of *capD* to bacterial virulence. As shown in Figure 8B, after 14 days of infection, mortality in the WT-infected group reached 75% (15/20) in contrast to 30% (6/20) in the Δ*capD*-infected group and 65% (13/20) in the CΔ*capD*-infected group. Mortality onset occurred 2 days post-infection in WT-infected frogs, with a median survival time of 8 days, whereas Δ*capD*-infected frogs started to die 3 days post-infection and persisted from day 10 until the end of the 14-day observation period without any mortality. Additionally, *capD* deletion reduced bacterial colonization in vivo. As shown in Figure 8A, the bacterial loads of the wild-type strain in the infected spleen and kidney were 4.67 × 10^5^ and 6.29 × 10^5^ copies/mg, respectively, and were 1.57 × 10^4^ and 5.92 × 10^4^ copies/mg in the liver and brain, respectively. However, the colonization level of the Δ*capD* mutant strain in the same tissues was significantly lower, at approximately 10^2^ copies/mg. The bacterial load in various tissues of the complemented strain was essentially consistent with that of the wild-type strain. No symptoms were observed in surviving frogs at the end of the experiment. In the PBS group, all frogs were in good condition. These results from animal experiments indicate that knockout of *capD* impairs the full virulence of *E. miricola* FL160902.

## Discussion

As a ubiquitous environmental bacillus, *Elizabethkingia* utilizes common capsular components that may confer resistance to antimicrobial agents and oxidative stress [26, 27]. In this study, we identified a typical CPS gene cluster containing the characteristic genes *wza*, *wzc*, and *wz*y based on the genome annotation information from the *E. miricola* strain FL160902. This CPS gene cluster is highly consistent with the one observed in Breurec’s study, exhibiting a highly conserved upstream region and a notably variable downstream region [3]. This variability in the downstream region may be responsible for the Wzy-dependent synthesis of polysaccharides with distinct chemical compositions corresponding to diverse capsular serotypes [3, 8]. The genetic organization within CPS gene clusters in *Elizabethkingia* genomes is strongly conserved among strains within the same phylogenetic sublineage [3]. Comparison of the CPS gene clusters from FL160902 with those of other *Elizabethkingia* strains revealed an intriguing correlation: *E. miricola* FL160902, which was isolated from a frog in China, shares the same type of CPS cluster with two African human-derived *E. anopheles* isolates (E27107 and E18064). The implications of these strains sharing the same CPS gene cluster warrant further investigation, particularly considering that capsular type differences in *Elizabethkingia* may influence immune cross-reactions and may be associated with distinct pathogenic properties. Moreover, the possibility of horizontal gene transfer in the CPS region between distinct sublineages requires further exploration.

The Wzy-dependent pathway, which is widely found in both Gram-negative and Gram-positive bacteria, is the primary and crucial mechanism for polysaccharide assembly [19, 20, 28]. Fully polymerized capsule polysaccharides are transported through the periplasm and outer membrane by a trans-envelope capsule translocation complex, which consists of two accessory proteins, Wzc and Wza [29]. Wzc, a member of the inner (cytoplasmic) membrane-periplasmic auxiliary (MPA) protein family, facilitates high-level polymerization and translocation across the inner membrane, whereas Wza, a member of the outer membrane auxiliary (OMA) protein family, forms a multimeric channel that enables polysaccharide translocation across the outer membrane [30, 31]. Additionally, Wza interacts with the periplasmic domain of Wzc, regulating capsular polysaccharide export by controlling the opening and closing of the Wza protein [29]. Consequently, the activities of Wzy, Wzc, and Wza are integral for capsule synthesis. Our study revealed that knockout of the *capD* gene led to increased expression of *wza*, whereas *wzc* and *wzy* remained unchanged. These findings suggest that the knockout of *capD* impedes normal CPS synthesis in bacteria, leading to the upregulation of the polysaccharide export gene *wza* to maintain sufficient CPS production. CapD affects the expression of Wza proteins, which are essential for the Wzy capsule formation pathway, indirectly influencing CPS assembly, transport, and extracellular fixation. However, further studies are needed to elucidate the specific mechanisms of action involved.

The *capD* gene, which is involved in polysaccharide biosynthesis, is one of the most highly conserved regions in the CPS locus of *E. miricola* FL160902. Our study revealed that, compared with the wild-type strain, the *capD* mutant strain presented capsule deficiency, with a 45% reduction in cell wall thickness. These findings indicate that the Cap*D* protein plays a crucial role in the efficient synthesis of CPS in *E. miricola*. However, the fact that the deletion of the *capD* gene did not result in a total absence of the capsule suggests the presence of alternative compensatory mechanisms. These genes may involve other genes within the capsule biosynthetic pathway that can partially fulfil the function of *capD*. Hydrophilic polysaccharides, which are the main component of the bacterial capsule, can store up to 90% of the water and provide moisture to bacterial cells under desiccation conditions [32]. In *E. miricola* FL160902, deletion of the *CapD* gene leads to an increase in the surface hydrophobicity of the bacteria and a reduction in their desiccation resistance.

Biofilm formation is often associated with extracellular polymers, which enable pathogenic bacteria to resist antibacterial agents, hydrogen peroxide disinfectants, and the host immune system, thereby promoting bacterial survival and infection [33, 34]. The initial stage of biofilm formation involves bacterial adhesion, wherein polysaccharides are implicated in promoting attachment to surfaces and enhancing biofilm stability, as evidenced by studies on *Enterococcus faecium* and *Acinetobacter baumannii* [19, 21]. However, in our study, the deletion of *capD* resulted in a significant increase in biofilm formation in *E. miricola* FL160902. Moreover, hydrophobicity and adhesion to EPC and bEnd.3 cell lines were also elevated, suggesting that *capD* inhibits biofilm formation, hydrophobicity, and adhesion in *E. miricola* FL160902. Similar findings have been reported in *Klebsiella pneumoniae*, *Vibrio vulnificus*, and *Escherichia coli*, where the capsular polysaccharide does not increase bacterial adhesion but instead impedes specific binding and time-dependent interactions between bacteria and inert substrates [35–37]. This discrepancy may arise from variations in extracellular polysaccharides, differences in cell surface hydrophobicity, and the various adhesion mechanisms employed by different bacterial species [37].

To investigate the impact of the *capD* gene on the pathogenicity of *E. miricola*, we utilized in vivo infection models in frogs. Our results revealed that the *capD* knockout strain resulted in a significantly lower mortality rate than did the wild-type strain. Invasion and colonization by pathogens in the blood and tissues of the host are key factors in the pathogenesis of infection. In our study, the bacterial load of the mutant strain in frog tissues was significantly lower than that of the wild-type strain. These findings indicate that CPS influences the virulence of *E. miricola*.

The interaction between bacteria and macrophages is also crucial in the host defense against bacterial infections, with macrophages typically responsible for pathogen phagocytosis [38]. Mouse macrophage RAW264.7 cells are very important, and multiple studies have examined their interactions with bacteria [39, 40]. In this study, compared with those in the WT group, the number of intracellular mutant bacteria increased significantly, indicating that *E. miricola* is more easily phagocytosed by macrophages after the *capD* gene is deleted and that CPS is an important anti-phagocytosis factor in *E. miricola*.

In conclusion, we demonstrated for the first time that inactivation of *capD*, which is involved in capsular polysaccharide formation, enhances biofilm formation and cell adhesion. Furthermore, the bacterial survival rates were reduced in response to complement-mediated killing, desiccation stress, oxidative stress, and macrophage phagocytosis. Compared with infection caused by the wild-type strain, both frog mortality and the level of systemic infection were significantly lower in the frog infection model. These results indicate that CapD contributes to the pathogenicity of *E. miricola* and suggest that CapD could be a potential target for new antibacterial therapies against *Elizabethkingia* spp.

## Supplementary Information

**Additional file 1. List of primers used for relative RT-PCR.**

**Additional file 2. Basic information of 28 genes in** ***E. miricola*** **FL160902 capsular polysaccharide gene cluster.**

## Data Availability

The datasets used and analysed during the current study are available from the corresponding author upon reasonable request.
