# Supplementary material for: capD deletion in the Elizabethkingia miricola capsular locus leads to capsule production deficiency and reduced virulence
Source: Vet Res. 2024 Nov 11;55:148. doi: 10.1186/s13567-024-01394-8 (PMC11552330; doi:10.1186/s13567-024-01394-8)
Supplement: Supplementary file 1 — Additional file 1. List of primers used for relative RT-PCR. [file 13567_2024_1394_MOESM1_ESM.docx]

| Primers | Sequence (5’-3’) | Product Size |
| --- | --- | --- |
| 16S-qPCR-1 | CGCTTAGTCTCTGAATCCTA | 234 bp |
| 16S-qPCR-2 | CGAACTGCCATTGATACTG |  |
| *capD*-qPCR-1 | TTAAGGCGCCATCTGTCCAA | 167 bp |
| *capD*-qPCR-2 | AGATCCTGCTCCTCCGGTAA |  |
| *wza*_1_-qPCR-1 | GAAGTGAGGGATTTGGTA | 262 bp |
| *wza*_1_-qPCR-2 | ACTGTATGTTGGCGATGA |  |
| *wza*_2_-qPCR-1 | TACCGAAACAGCACTCCG | 153 bp |
| *wza*_2_-qPCR-2 | TTCCCAGACATTTGTGGATT |  |
| *wzc*_1_-qPCR-1 | CGGTAAGAGGACGACAAG | 199 bp |
| *wzc*_1_-qPCR-2 | ATTACTTTCGGTGGGTGC |  |
| *wzc*_2_-qPCR-1 | CGACAGGGATTGCGGCTTAT | 166 bp |
| *wzc*_2_-qPCR-2 | GCTAATTCCAGCCCCGTCTT |  |
| *wzy*-qPCR-1 | TGCCATACAACTCAGAAG | 300 bp |
| *wzy*-qPCR-2 | GAGTGATACCCAAACAAG |  |

**Additional file 1. List of primers used for relative RT-PCR**

|  |
| --- |
|  |
