# Supplementary material for: capD deletion in the Elizabethkingia miricola capsular locus leads to capsule production deficiency and reduced virulence
Source: Vet Res. 2024 Nov 11;55:148. doi: 10.1186/s13567-024-01394-8 (PMC11552330; doi:10.1186/s13567-024-01394-8)
Supplement: Supplementary file 2 — Additional file 2. Basic information of 28 genes in E. miricola FL160902 capsular polysaccharide gene cluster. [file 13567_2024_1394_MOESM2_ESM.docx]

| **Additional file 2. Basic Information of 28 Genes in *E. miricola* FL160902 Capsular Polysaccharide Gene Cluster** | | | | | | |
| --- | --- | --- | --- | --- | --- | --- |
| Locus | Location | Length (bp) | GC% | Gene Name | Gene Description | Domain |
| GM001302 | 1368610-1369407 | 798 | 38 | *wza* | polysaccharide export family protein | Poly_export |
| GM001301 | 1366230-1368599 | 2370 | 34 | *wzc* | polysaccharide biosynthesis tyrosine autokinase | GNVR |
| GM001300 | 1365209-1366096 | 888 | 35 | - | DUF72 domain-containing protein | DUF72 |
| GM001299 | 1363885-1365153 | 1269 | 37 | *glyA* | serine hydroxymethyltransferase | SHMT |
| GM001298 | 1363339-1363806 | 468 | 28 | *recX* | RecX family transcriptional regulator | RecX |
| GM001297 | 1361218-1362948 | 1935 | 34 | *capD* | capsule biosynthesis protein CapD | Polysacc_synt_2 |
| GM001296 | 1360376-1361173 | 798 | 32 | *wza* | polysaccharide export family protein | Poly_export |
| GM001295 | 1357988-1360366 | 2379 | 32 | *wzc* | polysaccharide biosynthesis tyrosine autokinase | GNVR |
| GM001294 | 1356908-1357891 | 984 | 35 | *manC* | mannose-1-phosphate guanylyltransferase | NTP_transferase |
| GM001293 | 1355784-1356878 | 1095 | 26 | - | glycosyltransferase | Glycos_transf_1 |
| GM001292 | 1354601-1355800 | 1200 | 26 | - | glycosyltransferase | Glycos_transf_1 |
| GM001291 | 1353478-1354593 | 1116 | 25 | - | hypothetical protein | - |
| GM001290 | 1352230-1353477 | 1248 | 25 | *wzy* | O-antigen ligase family protein | Wzy_C |
| GM001289 | 1351171-1352226 | 1056 | 26 | - | glycosyltransferase | Glycos_transf_1 |
| GM001288 | 1350024-1351130 | 1107 | 30 | - | glycosyltransferase | Glycos_transf_1 |
| GM001287 | 1348897-1350027 | 1131 | 27 | - | glycosyltransferase | Glycos_transf_1 |
| GM001286 | 1347879-1348913 | 1035 | 35 | - | Mannose dehydrogenase | Polysacc_synt_2 |
| GM001285 | 1347433-1347846 | 414 | 31 | - | epimerase | FdtA |
| GM001284 | 1346318-1347436 | 1119 | 33 | *wbjC* | epimerase | Epimerase |
| GM001283 | 1345163-1346284 | 1140 | 33 | *wecB* | UDP-N-acetyl glucosamine 2-epimerase | Epimerase_2 |
| GM001282 | 1343947-1345155 | 1209 | 32 | - | glycosyltransferase | Glycos_trans_1_4 |
| GM001281 | 1343048-1343950 | 903 | 32 | - | NAD-dependent epimerase | Epimerase |
| GM001280 | 1342089-1343045 | 957 | 29 | - | glycosyltransferase | Glycos_transf_4 |
| GM001279 | 1341615-1342085 | 471 | 32 | *epsM* | hypothetical protein | Hexapep |
| GM001278 | 1340929-1341372 | 444 | 32 | *tagD* | adenylyltransferase/cytidyltransferase family protein | CTP_transf_like |
| GM001277 | 1340381-1340926 | 546 | 32 | *rfbC* | dTDP-4-dehydrorhamnose 3,5-epimerase | dTDP_sugar_isom |
| GM001276 | 1339294-1340373 | 1080 | 34 | *rfbB* | dTDP-glucose 4,6-dehydratase | GDP_Man_Dehyd |
| GM001275 | 1338387-1339253 | 867 | 38 | *rfbA* | glucose-1-phosphate thymidylyltransferase | NTP_transferase |
